# Applying Genomic and Bioinformatic Resources to Human Adenovirus Genomes for Use in Vaccine Development and for Applications in Vector Development for Gene Delivery

**DOI:** 10.3390/v2010001

**Published:** 2010-01-06

**Authors:** Jason Seto, Michael P. Walsh, Padmanabhan Mahadevan, Qiwei Zhang, Donald Seto

**Affiliations:** 1 Department of Bioinformatics and Computational Biology, George Mason University, 10900 University Blvd., MSN 5B3, Manassas, VA 20110, USA; E-Mails: jseto@gmu.edu (J.S.); mwalsh5@gmu.edu (M.P.W.); 2 Department of Biological Sciences, Vanderbilt University, Nashville, TN 37235, USA; E-Mail: padmahadevan@gmail.com; 3 Department of Microbiology, The University of Hong Kong, Pokfulam, Hong Kong SAR, China; E-Mail: zhang.qiwei@yahoo.com

**Keywords:** genomics, bioinformatics, molecular evolution, pathoepidemiology, field strain, adenovirus vaccine, human gene therapy vector, vaccine delivery vector

## Abstract

Technological advances and increasingly cost-effect methodologies in DNA sequencing and computational analysis are providing genome and proteome data for human adenovirus research. Applying these tools, data and derived knowledge to the development of vaccines against these pathogens will provide effective prophylactics. The same data and approaches can be applied to vector development for gene delivery in gene therapy and vaccine delivery protocols. Examination of several field strain genomes and their analyses provide examples of data that are available using these approaches. An example of the development of HAdV-B3 both as a vaccine and also as a vector is presented.

## 1. Introduction

Recombinant DNA technology has provided a molecular medicine scenario and opportunity where a disease or illness may be treated or prevented by the introduction of a specific gene or a fragment of a gene into the patient using a recombinant vector. This may be either as gene therapy, where a missing or non-functional gene is substituted with a functional version, or as vaccination, where a specific defined antigen is introduced [1,2]. Adenoviruses (AdVs) have been and are currently used as the basis for vectors delivering these genes [1–4], despite concerns of pre-existing immune responses [5,6]. In the way of understanding and perhaps avoiding these problems, a confluence of genomics and bioinformatics approaches is useful by providing the primary DNA sequence data and analyses of the original genomes from which the vectors are derived, allowing for a better understanding of the “starting material”- the genome- and its “expression”- the virus. These data also provide detailed relationships of the genes found within the genomes, as well as how these gene products may relate, computationally, to the antigens that may have been presented in the past, through HAdV infections, *i.e.*, the problem of pre-existing AdV immunity [5–7].

A contrasting, but related and also important, topic is the development of human adenovirus (HAdV) genomes and viruses as a vaccine against HAdV infections [8–10]. Epidemics are caused by the respiratory and ocular HAdVs, especially in large, concentrated and vulnerable, *i.e.*, unvaccinated, populations [11–17]. With their resulting high morbidity and occasional mortality, this preventable public health problem is amenable to a vaccine, similar to ones already produced, validated and deployed previously in the U.S. military basic trainee population [8–10,18]. The same concerns and ‘mechanical problems’ affecting the development, safety and wide-spread applications of these HAdV vaccines, which are based on the use of the genomes themselves as both vector and antigen, allow lessons learned, using genomics and bioinformatics, to be transferred to their applications as gene delivery vectors in gene therapy and in vaccine therapy. Hence, the HAdV genomes themselves will be the focus and context of this survey of the genomics and bioinformatics resources, which provide an information-laden basis for the rational design of HAdV vaccines and vectors for gene delivery.

There are two contrasting observations of the pathoepidemiology of these viruses that have important implications for both vaccine development and in its use as a gene delivery vector. First, HAdV is a pathogen that causes a range of human illnesses, including respiratory, ocular, gastrointestinal and metabolic diseases [19,13]; however, they may also infect aymptomatically [13]. Species C outbreaks have been reported as latent infections [20], which again may lead concerns of seroprevalence. This presents problems of pre-existing immune responses and the pathogenicity itself. Additionally, as the specific symptoms and illnesses are reflections of the particular organ or tissue infected, gene delivery roles are limited to the cell tropism of the particular HAdV.

Second, genome stability is an important consideration. Recently, there are two contrasting observations of genome stability that have been presented, at the genome sequence resolution level, for five recent field isolates (manuscripts under review or in press) and three emergent pathogens [14,21,22]. Coupled with the literature that multiple simultaneous HAdV coinfections have been reported as inseparable mixtures [23] and documented using molecular typing methods [24–27], concerns of possible genome transfer between the coninfectants, perhaps resulting in new strains and serotypes [23], are raised. These new strains may arise as recombinants, particularly under conditions that may be ideal, such as an immunocompromised individual [23,19,28]. Recombination has been suggested as a pathway for new serotypes [29]; and in general, recombination is reported for HAdV under different conditions [30–33]. This phenomenon has been taken advantage of in constructing vectors [34,35]. As an infectious disease pathogen, putative recombinants are reported as “intertypic” and “intermediate” [36–40], with epitopes mapped by serum neutralization and/or molecular typing methods. These techniques are limited and allow only a partial analysis. Recent whole genome and bioinformatic analyses document several recombinants as highly contagious emergent pathogens [38,14,41,21,22,17]. These observations suggest that, perhaps at a very low rate, recombination should be a consideration for the safety and efficacy of HAdV vaccines and use as vectors.

This is, and should be, balanced by a survey of several field strains which, along with their prototype genomes and other similar isolates, suggest that HAdV genomes may be stable in general, and, in one case of HAdV-C5, remarkably stable with four base changes across fifty years circulation both in the population and in a laboratory context (manuscript submitted).

### 1.1. Synopsis of HAdV biology

Adenoviruses are double-stranded DNA viruses that infect all vertebrates spanning fish to snakes to birds to humans [42,43]. Three recent reviews survey HAdVs as human pathogens, viruses and a “basic biology model subject” [19,13,44], so the reader is referred to these excellent sources for further detailed information. In brief, although their ca. 35,000-nucleotide genomes are relatively similar, there are many sequence differences (for example, species A is 58% identical to species C) along with differences in the proteomes encoded that are reflected by differences in their individual biology. These account for their tissue tropism, virulence, pathogenicity, host response/immune systems evasion and other biological characteristics. HAdVs are partitioned into seven “species” A–G, with species B separated into subspecies B1 and B2. Species G is recently recognized with the identification and description of a novel type 52 that differs from previously characterized and defined HAdVs, using genomics and computational methods [45]. Fifty-one serotypes based on serum neutralization and molecular typing techniques [23,13], were recognized until HAdV-G52 [19,13,44,45]. With, and based on, the genomic and bioinformatic analyses of several emergent HAdV pathogens from recent ocular and respiratory outbreaks, there are now 55 types reported in the literature [14,21,22]. As an aside, at the recent 9th International Adenovirus Meeting held in Dobogókő, Hungary (April 2009) where some of these genomic and bioinformatic data were presented, along with descriptions of two of the novel types, a formal “open floor” discussion led to a consensus of using “type” as part of the HAdV nomenclature scheme, allowing genome data to differentiate HAdV and keeping the original serotype names for 1–51. This has been touched upon in the literature earlier with opposing viewpoints [46]. As will be reported, and as suggested by a subcommittee at the meeting, genome data and computational analyses support and reconfirm the existing “serotype” nomenclature and classification in the context of the proposed “type” classification and nomenclature (manuscript in preparation).

### 1.2. Early genomics of HAdV

Despite the early recognition of the importance of HAdV as an infectious disease pathogen [19,13], its continued role as a globally circulating pathogen [13–15,38,26,17] and its previous role as a model “organism” in DNA replication biochemistry, cell and molecular biology [44], HAdV genomes and their analyses have lagged behind other genomes in the genomics era until recently. As DNA sequencing technologies have improved and as the focus of DNA sequencing targets shifted from smaller “feasible” genomes, e.g., Phi-X174 and mitochondrion, to the larger, e.g., *H. influenza* and *E. coli*, and then to the much larger, “more relevant” (to human health and well-being) and “sexier and exotic” genomes, e.g., human, rice, silkworm and panda, adenoviruses were left behind. These thoughts do “not hold water” as adenoviruses have a tremendous impact on human biology both as a pathogen and as a biotechnology tool! As of 2002, there were only five HAdV genomes archived in GenBank, consisting of genomes that were “cobbled together” as composites of earlier published data that were coupled with “final pieces” sequenced to put together a complete genome. The original sequences, deposited by different researchers, were obtained using different sequencing methodologies and using, likely, in-house laboratory-circulating version of the prototypes. These include genomes for HAdV-C2 [47], HAdV-C5 [48], HAdV-A12 [49], HAdV-D17 (Genzyme Corp.; GenBank 1998) and HAdV-F40 [50]. With the exception of HAdV-D17, having documented sequencing errors [43], these genomes are still useful as reference sequences and relevant to understanding HAdV biology and evolution, especially with the continued annotation of their genomes [43] and the recent high resolution descriptions of recombination events in HAdV genomes [41,51,21,22]. The importance of the HAdV-C5 genome is demonstrated in the resequencing of its genome using a single methodology, and by the preparation, designation and distribution of an industry standard, “Adenovirus Reference Material” (ARM), available from ATCC (Manassas, VA) [52].

### 1.3. Current genomics of HAdV

In contrast to the “tsunami” of genome sequences generated for bacteriophage, viral, mitochondrial, bacterial and eukaryotic genomes paralleling the rapid and continued development of faster, robust, more efficient and more cost-effective DNA sequencing technologies and its application to many organisms and groups of organisms, the HAdV genomes database had been sparsely populated. Given the increasing and improving number of genomic and bioinformatic tools and methods, it seemed sensible to examine the well-studied AdVs using these approaches. In particular, one application is in biotechnological applications including vaccine development and diagnostics platforms. Apparently this was of interest to several research groups as well, as a “seiche” (rather than a tsunami) of HAdV sequences appeared. HAdV-B35 was sequenced independently by two different groups, both interested in vector development and the use of HAdV-B35 as an alternative to HAdV-C5 in gene therapy and gene transfer applications [53,7], and, in part, to bypass pre-existing HAdV-C immunity. The HAdV-B11 genome was sequenced independently twice at this time as well [54,55]. In addition, the first HAdV to be described clinically and historically was also sequenced in this time period, as a prelude to sequencing and analyzing the rest of the prototypes and several field strains responsible for respiratory diseases, especially acute respiratory disease (ARD) [56]. A practical consideration for these genomes was for the identification of sequence diagnostic probes for the development of a microarray-based surveillance and diagnostics assay [25]. Subsequently, the release into GenBank of 16 additional genome data sets from the authors, HAdV-B3 (two genomes), HAdV-B7 (three genomes), HAdVB-16, HAdV-B21, HAdV-B50, HAdV-B14, HAdV-B34, HAdV-C5, HAdV-C6 (embargoed) and HAdV-E4 (four genomes), added to the growing number of available HAdV genomes [25,56–60]. Also available were five chimpanzee AdV genomes [61,62], which are of interest as alternative gene delivery vectors. Currently, there are 31 prototype and 37 field isolate genomes deposited in GenBank, with the number expected to grow as clinical investigations of HAdV-associated illnesses are leading to the identification of putatively interesting HAdVs. One such interesting observation involved a recent fatal case of ARD, with an identified HAdV isolate that also was a highly contagious ocular pathogen [63].

## 2. Tools and methodologies of bioinformatics for adenovirus genomes

### 2.1. Genomics: acquisition of data

The advent of genomics and bioinformatics has complemented and extended the available data and tools for characterizing and developing vaccines, as well as for vector development in gene transfer and delivery based on HAdVs. Having in hand the exact nucleotide sequence allows more precise and defined manipulation of the genome. Knowing exactly where and how much of the original genome to delete to allow the insertion of expressible heterologous sequences and having rapid access to the set of in silico proteome allows comparisons across similar genomes to ascertain critical and “unimportant” genes, and allows non-essential genes to be deleted. Limited resequencing ensures no genome and gene insert changes are inadvertently in the final product. Finally, as cell tropism is embedded in the primary sequence and plays a role in the delivery of genes to targeted cell types [64–66], it may be possible to alter tropism.

Sanger-based dideoxy-sequencing chemistry has been the standard methodology to date. Its several variations, conveniently and uniformly converted into kit formats, allow for high-throughput automation; for example, DYEnamic ET Terminator Cycle Sequencing kits (Amersham Biosciences; Piscataway, NJ, USA) generated ladders that were resolved on an ABI Prism 377 Sequencer (Applied Biosystems; Foster City, CA, USA), and were the basis for the genomes sequenced by the authors. The development and application of “Next Generation” instrumentation and protocols are creating opportunities to obtain much greater numbers of HAdV genomes, allowing for detailed examinations of HAdV, including pathoepidemiology and molecular evolution. Given the number and flux of new technologies, and the lack of use with these systems in AdV genomic studies, we will not discuss their use here. It is noted that each technology has its own particular strengths and weaknesses, and these will need to be understood with regards to AdV genomics.

In general, regardless of the technology, there are two common considerations for HAdV genome sequencing. One is accuracy and quality control. For the Sanger-based method, a minimum three-fold coverage across the entire genome is required, with problematic regions and potentially relevant SNPs to be covered by additional re-sequencing. A “2+1” strategy, that of obtaining sequences comprising both strands, allows high confidence in the genome data. Another method to ensure high quality sequence data is to analyze the proteome with computational means. For all sequencing methods, sequence assembly, particularly from “short read” ladders of some protocols, and the quality control of these genome sequences may be augmented by the annotation process. In other words, there are essential genes, such as hexon, penton, fiber, and genome features, such as the inverted terminal repeats (ITRs), that must be present and within a certain conserved range of sequences. Having an annotation allows unreliable data to be re-sequenced for resolution, allowing a ten-fold increase in sequence accuracy.

Second, there are regions that may be difficult to sequence, given a particular technology. One example for the Sanger methodology is the ends of the AdV genome. Both ends of this linear HAdV molecule contain inverted terminal repeats (ITRs). These should complement each other, and serve as a quality control check. One end may be sequenced by DNA polymerase “running off the template”; the other end is problematic, as DNA polymerase requires a template to initiate. An additional complication is that HAdV genomes contain a covalently linked protein that is attached to the end. One solution to circumvent these obstacles is to use a “rapid amplification of cDNA ends” kit (5′/3′ RACE; Roche Diagnostics Corp.), with modifications [22].

### 2.2. Bioinformatics

#### 2.2.1. Genome analysis

Computational analyses of the genome involve examining the nucleotide sequence of the genome and the protein sequences of the proteome. There are many software tools available, both as commercial packages and as public resources on the Internet. One site housing the URLs for many public resource software tools is http://molbiol-tools.ca/. Specific tools used in our studies are described here and detailed in Table 1.

For DNA sequencing ladders assembly, DNA Sequencher (Gene Codes Corp.; Ann Arbor, MI, USA) was used for the completion of the seventeen genomes noted earlier as submitted by the authors. This software can also be used to align sequences and, importantly, manually move them, allowing visual characterization of recombinant or deleted sequences.

As noted above, once a consensus contig genome is produced, a quick examination of certain “landmarks” is useful, for example, the ITRs, pTP and Pol genes are difficult to sequence. A first pass annotation, to be described later, is recommended as a sequence quality control step before additional effort and excitement are expended, or as admonished by a biochemist as one of the “Ten commandments of enzymology”: “Don’t waste clean thinking on dirty enzyme” [67].

The attributes of the genome include GC content, with the percent GC diagnostic of species: A (47%), B1 (51%), B2 (49%), C (55%), D (57%), E (57%), F (51%) and G (55%). Genome lengths are not indicative of species and range from 34,125 bases (HAdV-A12) to 36,015 (HAdV-E4). The percent identity of a new genome may be determined relative to sequenced genomes; for example, species A is 58% identical to species C. The genome nucleotide sequence can be examined for repeats using PipMaker. This software uses a BlastZ algorithm to compute the local alignments of pairs of genomes and produces dot plots that give an indication of the similarity of the two genomes, as well as highlights any genome rearrangements [68]; the highest similarity scoring fragments will align on a diagonal. On the same site, zPicture gives another version of this genome identity analysis.

Multiple whole genome alignments, of genomes the size of HAdV, can be made using MAVID (http://baboon.math.berkeley.edu/mavid) [69]. MAVID, in turn, produces alignment outputs that may be ported into phylogeny tree analysis algorithm. For our studies, neighbor-joining trees [70] are constructed using MEGA4 [71]. Genome recombination events can be found in the genome alignments. Recombination is a contributing factor in the evolution of HAdV [14,51,21,22], as noted for driving serotype evolution based on serum neutralization studies [29]. Sequence recombination can be detected whole and partial genome alignments, using Bootscan and SimPlot [72], with other comparable software also available [73].

Although seemingly anachronistic, restriction enzyme (RE) pattern analysis is still very useful, particularly to understand the context of the genomes relative to previously reported RE patterns of isolates reported in the literature. It should also be noted that as a “whole genome scan” tool, *i.e.*, a “genotyping” tool, RE patterns are effective for a rapid visual overview and comparison of the nucleotide genomes. The pDRAW32 software is one example for this in silico RE analysis. Also, the availability of genome data allows unlimited RE patterns and resolution of “faint” and multiple bands, and allows much better resolution than gel-based and photograph-based gel data in the literature.

#### 2.2.2. Proteome analysis

A full-length annotation of coding and non-coding sequences completes the presentation of the genome sequence and extraction of information from the nucleotide string. In the past, HAdV and simian adenovirus (SAdV) sequences deposited in GenBank were incompletely annotated, with only a minimal annotation associated, particularly if submitted for patent purposes. We have developed a beta version of an automated genome annotator for our studies. This gives a “first pass” annotation that is suitable for assessing sequencing data quality. Refinement of the annotation manually as well as the examination of genome differences can be done using a genome viewer such as Artemis [74].

The proteome may be examined computationally, using percent identity comparisons of the nucleotide sequence and the amino acid percent identities of the proteins. These are manually calculated using the EMBOSS package [75]; more recently a beta version of an automated tool allows the same calculations. This provides an independent view to any recombination events.

Individual proteins and genome landmarks, e.g., ITRs, may be analyzed phylogenetically, using CLUSTAL for multiple sequence alignments (MSA) [76] and porting into phylogeny tree analysis software. One example of the relevance of this approach is a report showing the zoonotic origin of HAdV-E4 from chimpanzees [57]. An implication of the proteome analysis is a suggestion that the use of chimpanzee AdVs as alternatives in gene delivery in order to bypass pre-existing immune response may not be advisable, or should be done with caution.

#### 2.2.3. Informatics support

**Figure 1.**
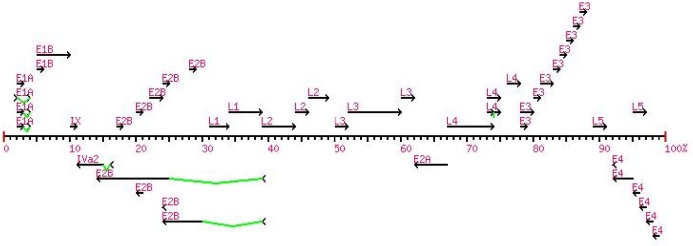
Automated gene-mapping tool. A field strain of HAdV-B7 (accession number AY601635; strain designation #NHRC 7151) has been sequenced and annotated. Its coding sequences are displayed using a gene-mapping tool using a derived annotation table.

The Internet provides opportunities for worldwide interactions and collaborations, and for community-based resources. An “AdenovirusWiki” has been developed as an open resource for adenovirus research. In addition to the software tools available on the Internet, several local tools are also available, as beta versions: automated genome annotation; proteome percent identity analysis; and gene mapping tool (Figure 1); (www.irgolf.com/genemapv2). These provide for a pipeline to take a genome nucleotide sequence through analyses to produce genome annotations, proteome identification and analyses and a presentation of the coding sequences on a genome schematic.

A local tool developed in the authors’ research group is “Virus Genome Annotation Tool” (VGAT). This is a beta version that is publicly available, with the caveat that it is a test version, at http://binf.gmu.edu/zenith/tool/lghmms.php. VGAT uses “Hidden Markov Models” (HMMs) to annotate virus genomes. Currently, this software tool has been trained to annotate members of the HAdV-D species. It can be expanded to include members of other HAdV species, and the ability to add user defined training sets will be added.

A tool for automatically comparing the protein percent similarities in proteomes relative to their homologs in other proteomes is available in a beta form. The protein alignments and percent identities are calculated using a BioJava implementation [77] of a Needleman and Wunsch algorithm. When completed, the tool will be publicly available via the Internet; currently this tool is available upon request.

#### 2.2.4. Bioinformatics Tools Summary

Again, all of the computational tools noted for our studies are summarized in Table 1. As mentioned in the text, two are beta versions and need to be optimized. There are additional and equivalent software tools available over the internet, including multiple independent tools for similar analyses. A caveat is that some may be limited to certain computer platforms and need to be compiled. In some cases, the original contributors may no longer support some tools (orphans); however, some tools may be very useful and still supported, albeit at a different URL due to the contributor changing physical addresses. These may be found by ‘googling’ the tool name and/or the author on the Internet to locate the tool.

## 3. Considerations of HAdVs for vaccine development and for vectors development for gene transfer and delivery

Limited molecular typing, e.g., PCR amplification coupled with DNA sequencing of certain targets, is a quick informative method to be applied in rationally designing adenoviral gene delivery vectors and in screening HAdVs and constructs as vector candidates [78–80]. Molecularly typing the outer coat proteins, hexon, penton and fiber, is important as they have critical roles in tissue tropism as well as in the host immune response to the virus. A caveat is that genome recombination may occur at other locations that may have subsequent and important consequences in the biotechnological application of the genome.

Although HAdV species C was initially the focus as vectors for biomedical and biotechnological applications, current interest range beyond this group. There have been many vectors based on other human, and even non-human, AdV serotypes that have developed as vectors for gene delivery and vaccine development. One review by Stone *et al*. [81] is an example. Primary literature citations include [2,4,7,53,62,82–89]. It is anticipated that genomics and bioinformatics resources will aid in these on-going work and development.

### 3.1. Natural variation of HAdV genomes

As noted earlier, recombination events are hallmarks of HAdV *in vitro*, and have now been documented in whole genome studies. The rate of recombination is not yet known. It may be that some species or types may be amenable to recombination based on sequence, e.g., hotspots, and biology, e.g., cell tropism and coinfection. Mutations as nucleotide changes, such as insertions and deletions (indels) and substitutions, are more common. However, given the fidelity of the DNA polymerase, the relevance of these genome changes remains to be elucidated. The question relevant to both HAdV vaccine development and gene delivery vector design is: How stable are these genomes?

**Figure 2.**
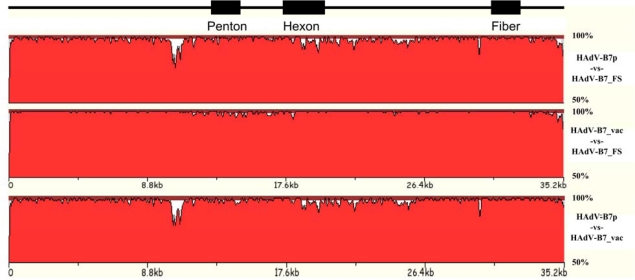
HAdV-B7 genome comparisons. Three HAdV-B7 genomes, spanning ca. 1950s to 1990s, are analyzed using zPicture. Locations of the outer coat proteins, which are critical putative epitopes and cell tropism determinants, are arrayed for reference.

Genomes from some isolates appear to be very stable, at least from the viewpoint of their antigenic epitopes. For example, the HAdV-B7 and E4 vaccines were highly effective in the U.S. military basic trainee population [8–10,18] for over twenty-five years [9]. This suggests, at least, a conservation of the epitopes for these two serotypes. A genome comparison of the prototype *versus* the “vaccine” (presumably the “then-circulating” and dominant) strains of both HAdV-B7 and HAdV-E4 showed few genome changes, mainly indels and base substitutions [57–59]. These strains were of the 1950s and 1960s era. A pair of more recent HAdV-E4 field strains (accession number AY599837; strain designation #NHRC 3) and (AY599835; NHRC 42606), from two different outbreaks, and a recent HAdV-B7 field strain (AY601634; NHRC 1315) were sequenced, as 1990s isolates, to allow a comparison of their genomes. These showed similar limited mutations as well. Figure 2 displays whole genome comparisons of the three HAdV-B7 strains, across approximately forty-five years. Based on these whole genomes analyses and based on molecular typing, e.g., PCR amplification coupled with sequencing, of critical epitopes, it is likely the vaccines in production will be effective, as these genomes appear relatively stable.

Proteome analysis allowed a detailed examination of all three HAdV-B7 genomes. Table 2 shows that despite the nucleotide differences, the protein percent identities are high, with the exception of the agnoprotein and the E3 7.7 kDa protein. The E3 difference may be important as those proteins may have roles in host immune response [90].

**Figure 3.**
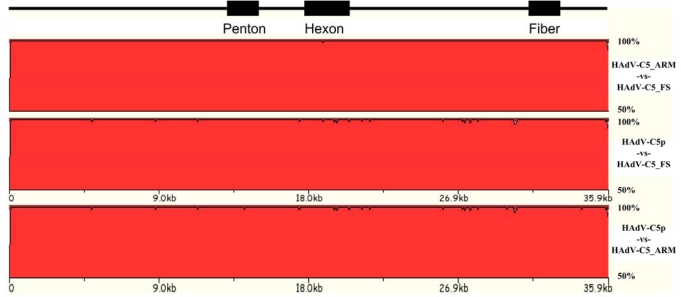
HAdV-C5 genome comparison with zPicture. Genomes of a laboratory-circulating strain and a coinfectant field strain of HAdV-C5 are compared with the reference prototype (ARM). The field strain genome has ∼100% sequence identity with the ARM strain, with four nucleotide differences across 45 years.

Similar genome and proteome data were obtained from analyses with HAdV-E4p, HAdV-E4_vac, HAdV-E4_FS1 and HAdV-E4_FS2 (data not shown). Two other prototype (ca. 1960s) and field strain (ca. 1990s) genomes have been paired and sequenced (or extracted from GenBank) as well: HAdV-B3 and HAdV-B3_FS (AY599836; NHRC 1276); and HAdV-C5 and HAdV-C5_FS (AY601635; NHRC 7151). Additionally, the HAdV-B3 genomes were compared with two field strains sequenced and described in China as well as a laboratory-circulating strain [91]. Genomes available for the HAdV-C5 analysis are even more interesting, as two prototype genomes were available from GenBank: 1) the original report, a composite presumably of several laboratory-circulating strains; and 2) an amplification of the original prototype from ATCC (Manassas, VA, USA) and now available as an “Adenovirus Reference Material” (ARM). Shown in Figure 3, these versions of the prototype were compared to the genome of a field strain, HAdV-C5_FS, which was isolated as one of a pair of coinfecting HAdVs (manuscript submitted). The other coinfectant was HAdV-B21 and no signs of recombination in the HAdV-C5 genome were observed. Only four genome changes (one substitution and three indels) separated the 1998 field strain from the 1953 prototype (ARM). HAdV-C5_FS differs slightly more from the circulating laboratory strain (99.9%), suggesting laboratory passages allow some unselected mutations to accumulate.

The apparent stability of these examples suggests that, in some cases, HAdV genomes are not as vulnerable to large-scale genome changes, such as recombination events. Accumulation of indels and base substitutions do occur, as would be expected, although in one case, HAdV-C5, it can be surprisingly few in number. All of these observations, including the highly effective nature of the HAdV-B7 and E4 vaccines earlier, imply that vaccines developed and vectors developed using HAdV genomes may be stable and useful for a period of time.

### 3.2. Natural variation of HAdV genomes: new types, new species, and vector candidate

Novel HAdV may be candidates in the quest for effective, appropriate (e.g., tissue and organ specific) and safe (e.g., asymptomatic and non-immunogenic) vectors. Genomics and bioinformatics have provided the identification and characterization of HADV-52, isolated from the stool of a patient with gastroenteritis [45]. As it was distinguished on the basis of genomics and bioinformatics rather than the traditional immunochemical techniques, it is referred to as “type” rather than the inappropriate “serotype”. In addition, the case has been made for it as both a new type and a new species as well; the opposing view has been discussed in the literature [46]. There are additional computational data, derived from bioinformatic methods noted earlier, that were not reported in the original report which provide strong support for this as well. HAdV-G52 shows a very high whole genome percent nucleotide identity with SAdV-G1, a simian (monkey) AdV, at 95.5%. This has been proposed as a member of species G. In contrast, the percent identity with SAdV-G7 is also high at 82.9%, another proposed species G member. The percent identities between the next phylogenetically closest, and also gastrointestinal, viruses HAdV-F40 and HAdV-F41 are much lower at approximately 69%. For reference, the percent identity between HAdV-F40 and HAdV-F41 is 85.8%, both members of species F. GC analysis shows a clustering of HAdV-G52 (55.1%), SAdV-G1 (55.2%) and SAdV-G7 (56.3%) as opposed to HAdV-F40 (51.2%) and HAdV-F41 (51.0%); again, GC contents seem to correlate with species grouping when surveyed across all of the sequenced genomes (data not shown).

**Figure 4.**
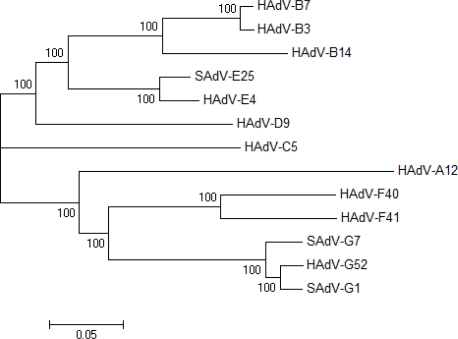
Whole genome phylogenetic tree. HAdV-G52 subclades with SAdV-G1 and SAdV-G7. HAdV-F40 and HAdV-F41 subclade separately. Numbers presented are percent bootstrap replicates.

Moreover, whole genome phylogenetic analysis (Figure 4) shows that HAdV-G52 subclades with SAdV-G1 and SAdV-G7. HAdV-F40 and HAdV-F41 forms a separate subclade. Proteome analysis, in the form of percent similarities, also shows closer relationships between the proposed “G” species members than with the other HAdV (data shown in Table 3). HAdV-G52 is missing the RL3 protein, which is unique to HAdV-F proteomes; this is also not in genomes of the other available genomes including the two simian ones grouped as species G. The RL3 protein is a 6.7 kDa protein that is encoded by E3 [92]. The function of RL3 is thought to be in directing glycoproteins to the endoplasmic reticulum [92].

These results, taken with the original data, strongly suggest that HAdV-G52 is more related to SAdV-G1 and SAdV-G7 than to HAdV-F40 and HAdV-F41, and sufficiently different to merit designation as a new type and species. It would be inappropriate to refer to this strain as a “serotype” HAdV-G52 as no comprehensive serotyping data were provided to distinguish it from the accepted ones; however, it is clearly different from the 52 established serotypes. These data also suggest the two monkey AdVs are likely members of species G and may suggest a zoonotic origin for HAdV-G52. Given the lower percent identity scores with the other HAdVs (data not shown), HAdV-G52 may be a strong candidate as a vector for gene delivery, possibly avoiding pre-existing immunity issues.

### 3.3. Natural variation of AdV genomes: non-human primate AdV genomics and vector candidates

The persistence, infectivity and wide distribution of HAdV in general lead to concerns of pre-existing immunity, as characterized by seroprevalence [7]. This, in turn, leads to concerns with the use of HAdV as vectors. In an attempt to develop vectors that may be free of potential problems, alternative non-human AdVs may be appropriate substitutes as vector candidates, especially those from the great apes which presumably can infect human cells due to similarities.

A growing number of such AdVs are beginning to be isolated, characterized and examined for use as vectors. A recent contribution of 33 novel non-human primate genomes (30 ape and three macaque) has been reported and deposited in GenBank [93]. These are in addition to several monkey AdV genomes, sporadically deposited as simian AdV (SAdV) since 2004, and the five original chimpanzee AdV genomes, also noted as SAdVs, deposited into GenBank in 2004 [61,62]. The first chimpanzee AdVs were originally deposited at the American Type Culture Collection (ATCC) in the 1960s-70s, so these 33 additional genomes represent a recent, renewed and directed interest in novel non-human primate AdVs. Biotechnological applications, including vector applications, appear to drive the enthusiasm for the collection and characterization of these genomes. At least three groups are contributing to this seiche of monkey and great ape AdV genomes (noted collectively in the past as “simian”), as per several reports at the recent 9th International Adenovirus Meeting (Dobogókő, Hungary; April 2009). Given the wide diversity of genomes and this larger collection, it was suggested then by one of authors that it may be appropriate to standardize the nomenclature, to one that is also discriminatory and informative. Rather than classifying them all as “simian” AdVs (SAdVs; for example, [93]), subclassification into chimpanzee (ChAdV; for example, [82]), bonobo (BoAdV), gorilla (GoAdV) and perhaps monkey (MoAdV), *etc*. may be more appropriate, especially for eventual “Big Picture” analyses of all genomes and for discussions of HAdV origins, molecular evolution, natural histories, taxonomy and virus reservoirs.

As a result of recent interests, the inventory of non-human primate AdV genomes is growing at a rapid pace, and the genomes are providing alternative biotechnology tools as well as providing resources for a more detailed glimpse into the biology, genomics and bioinformatics of HAdVs. The availability of these and other primate genomes allow more thorough computational analysis and finer resolutions of earlier observations. For example, discussed earlier were the zoonotic origins of HAdV-E4 and species E from the chimpanzee [94,95,57]. The recent 33 novel non-human primate genomes are parsed by genome analysis into HAdV species B, C and E [93], complementing the B and E species partitioning of the original SAdV-21 through SAdV-25, and confirming a close phylogenetic relationship with the HAdVs. As the recently described AdVs were collected as samples from substantial and persistent shedding in the stools of asymptomatic and apparently healthy primates, a comment (and caution) as to a zoonosis potential was noted. Noted also was the possibility and observation of intraspecies recombination in one of these primate AdV genomes [93], echoing recent reports of genome recombination in HAdV [14,51,21]. These two possibilities, zoonosis and recombination, may have relevance in understanding pre-existing human seroprevalence as well, as the earlier computational analysis of HAdV-E4 showed protein homologies to the chimpanzee AdVs (SAdV-21 through SAdV-25). Further bioinformatic analyses should be applied to these new genomes.

Chimpanzee AdVs have been developed into vectors for potential human applications recently and in the near past [82–84]. It is anticipated that both genomics and bioinformatics will play large roles in the further and continuing development and applications of these AdVs of human uses, especially within the context of the rational design of vectors for gene delivery.

## 4. Applications

Genomics and bioinformatics have provided a more detailed understanding and another dimension of these viruses through their genomes and proteomes. These are very useful in the context of vaccine development and also in the continuing biotechnological development of HAdV genomes as a vector for gene delivery. As an example, HAdV-B3 is discussed as a subject for both applications. This particular ARD infectious disease agent remains a global pathogen and is a public health problem, particularly in high-density populations [96]. Data suggest that a vaccine developed against a particular HAdV may be cost-effective and will be an effective prophylactic for an extended period of time, e.g., stable genome, similar to the original HAdV-B7 and HAdV-E4 vaccines. On the other hand, seroprevalence due to its circulation is a concern and limits its use as a vector [7].

### 4.1. Applications: development of HAdV-B3 vaccine

HAdV-B3 remains an important human pathogen for ARD [13,96]. The serotype is considered highly virulent and has been associated with high morbidity, due to pharyngoconjunctival fever and residual lung damage, as well as mortality in children [97,98]. Currently there is no effective vaccine against HAdV-B3 infection. Therefore, it makes sense to have a safe, effective, readily available and inexpensive vaccine available against this virus in the countries with very dense and vulnerable populations, especially in populations where the prevalence of HAdV-B3-specific neutralizing antibodies is very low. To this end, Zhang *et al.* have developed a replication-competent recombinant HAdV-B3 rAdΔE3GFP vector expressing eGFP as a vaccine candidate [85].

**Figure 5.**
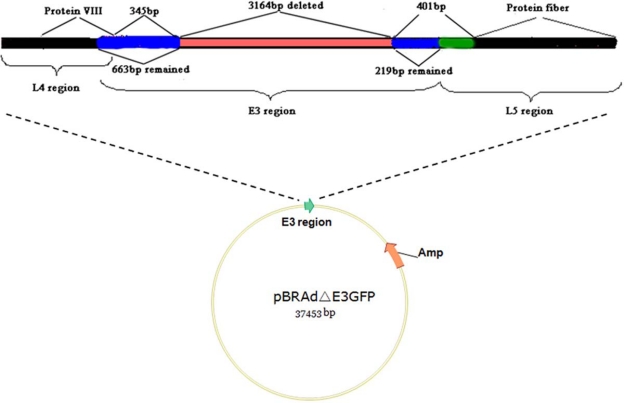
Deletion map of the E3 region in the recombinant pBRAdΔE3GFP vector. The red region indicates the deletion in the HAdV-B3. Blue regions indicate remaining left and right regions of the E3 region. Black regions indicate VIII protein and the fiber, L4 and L5 regions respectively. The green indicates the gap between the E3 region and the L5 fiber region.

As shown in Figure 5, a recombinant virus was constructed by deleting a 3,164 nucleotide segment in the non-essential E3 region (nucleotides 27,737–30,900), yielding the rAdΔE3GFP genome [85]. A CMV-eGFP-SV40 expression cassette (1,616 nucleotides) was inserted into the E3 region by recombination. The left 663 bp and right 219 bp flanking E3 regions remained in place. In theory, a maximum size of 4,800 nucleotides (foreign gene) can be inserted into this E3-deletion vector. Mice immunized with the recombinant eGFP AdV by either intramuscular injection, intragastric or intranasal inoculation routes raised a significant antibody response to eGFP and to the wild-type HAdV-B3 GZ1 strain at the same time [96,85]. Alternatively, Li *et al.* constructed another replication-defective HAdV-B3 by the deletion of the entire E1 region for use also as a vaccine candidate [86]. Wild-type HAdV-B3 can be neutralized by the sera from the mice intramuscularly immunized with this recombinant virus [86].

In the U.S., deployment of live enteric-coated oral vaccines against HAdV-B7 and HAdV-E4 was successful in removing both pathogens as agents for ARD for approximately twenty-five years [99]. Both vaccine strains presumably and selectively infected the lower intestinal tract, as administered in these enteric-coated capsules, and stimulated the production of appropriate neutralizing and circulating antibodies. No adverse signs or symptoms of illness were associated with these two vaccines [8,100]. Similarly, the replication-competent HAdV-B3 may act as an effective and safe vaccine candidate as well when administered in enteric-coated oral capsules. No helper cells are needed and the necessary virus titers should be easy to obtain. Additionally and importantly, this vaccine genome could be used either as a bivalent or trivalent vaccine for the delivery of more viral antigens. Continuing work with the heterologous expression of the HAdV-B7 hexon in this vector is underway [85].

### 4.2. Applications: development of HAdV-B3 as a vector for gene delivery

Many gene therapy vectors, to date, used in human clinical applications are currently based on species C members: HAdV-C5 and HAdV-C2. However, the apparent pre-existing immunity against them, from previous infections [101,7], and the lack of the coxsackie and adenovirus receptor (CAR) or integrin expression in target cells may be of concern, for the safety of the patient and the efficacy of these species C-based AdV vectors [64,66], respectively.

As a consequence and an attempt at rational design of vectors, species B have been explored as alternatives, both as to increase the range of cells infected and to bypass pre-existing immunity [53–55,87,81,7]. For example, HAdV-B3 is reported to gain entry into cells through alternate receptors: CDX, CD46, CD80 or CD86 [65,102–106]. These are expressed in a multitude of cell types, including important gene therapy target cells that express either no or low levels of CAR. Therefore, HAdV-B3 may be an alternative to HAdV-C5-based gene-transfer vectors. A recombinant E1-deleted HAdV-B3 vector has been engineered on a bacterial artificial chromosome [88]. It is efficiently transduced into CD46-positive rodent and human cells. Another replication-defective HAdV-B3 vector was also constructed independently by molecular cloning [86]. These viruses were shown to replicate in an E1-complementing cell line. Other recombinant species B-based replication-defective vectors have also been developed: HAdV-B7 [89], HAdV-B11 [107,87] and HAdV-B35 [53,7]. However, seroprevalence due to presumably previous HAdV-B3 infections may be of concern. More studies are still needed for the effective and appropriate safe applications of these vectors for *in vivo* gene transfer.

## 5. Conclusions

Taking advantage of the recent and continuing improvements in high-throughput DNA sequencing technology and methodology, coupled with a myriad of bioinformatic tools developed for other organisms and areas of research, the HAdV researchers now have a wealth of genome and proteome resources to apply to understanding the comprehensive and integrated biology of the virus, including deeper and finer points concerning viral origins, putative reservoirs, molecular evolution, natural histories and taxonomy. These data have been applied to the development of vectors for gene delivery, either for gene therapy applications or for the delivery of antigens in vaccine development. Several of the early genomes sequenced were done so for this purpose. Research examining the natural variation of HAdVs, as well as the molecular evolution of their genomes, particularly in the context of emerging pathogens, has shown that the genomes are seemingly stable, that is accumulating indels and base substitutions commonly and recombination less commonly. These observations have relevance in understanding the biology and the pathoepidemiology of adenoviruses as a whole, and, importantly, also have relevance in the development of vaccines against these pathogens as well as the biotechnological applications in vector development.

## Figures and Tables

**Table 1.** Summary of Bioinformatics Tools.

**Tool**	**Purpose**	**Availability**

Sequencher	sequence assembly	Commercial
PipMaker	genome sequence analysis	http://pipmaker.bx.psu.edu/cgi-bin/pipmaker?basic
zPicture	genome sequence analysis	http://zpicture.dcode.org
MAVID	whole genome alignment	http://baboon.math.berkeley.edu/mavid
MEGA4	alignment viewer, phylogeny	http://www.megasoftware.net/
Simplot	recombination analysis	http://sray.med.som.jhmi.edu/SCRoftware/simplot/
pDRAW32	*in silico* restriction enzyme digest	http://www.acaclone.com/
Artemis	sequence viewer, annotation tool	http://www.sanger.ac.uk/Software/Artemis/
EMBOSS	sequence analysis	http://emboss.sourceforge.net/
Auto % Id *beta*	sequence % id	available upon request
Clustal	sequence alignment	http://www.clustal.org/
Adenovirus Wiki	repository of adenovirus data	http://www.binf.gmu.edu/wiki/index.php/Main_Page
Mapping Tool *beta*	create gene maps	www.irgolf.com/genemapv2
VGAT *beta*	automated virus genome annotation	http://binf.gmu.edu/zenith/tool/lghmms.php

**Table 2.** Proteome Analysis of HAdV-B7. Percent identities of proteins amongst the reported three genomes of HAdV-B7 are calculated, relative to the HAdV-B7_FS proteome.

**HAdV-B7_FS**	**HAdV-B7_vac**	**HAdV-B7p**

E1A 28 kDa protein	98.9	99.6
E1A 32 kDa protein	98.3	99.6
E1A 6 kDa protein	98.3	100.0
E1B 20 kDa protein	100.0	98.3
E1B 55 kDa protein	100.0	99.0
IX protein	100.0	100.0
IVa2 protein	99.3	98.9
DNA polymerase	99.7	98.2
Hypothetical	99.1	98.1
agnoprotein	99.5	72.7
Hypothetical	100.0	68.4
Terminal protein	100.0	98.8
Hypothetical	99.2	95.5
Hypothetical	98.9	93.4
52 kDa protein	99.2	97.4
IIIa protein	99.1	99.7
penton base protein	98.7	99.3
VII protein	98.4	99.5
V protein	98.3	98.3
X protein	98.3	98.3
VI protein	96.4	96.8
hexon	99.8	97.0
protease	100.0	97.6
DBP	99.8	97.7
100 kDa protein	99.6	98.2
33 kDa protein	98.3	83.9
22 kDa protein	100.0	97.5
VIII protein	86.8	86.3
E3 12.1 kDa	100.0	100.0
E3 CR1-α	100.0	99.3
E3 glycoprotein	100.0	100.0
E3 CR1-β	95.5	93.3
E3 CR1-γ	99.5	98.4
E3 7.7 kDa protein	100.0	59.1
E3 RID-α	100.0	100.0
E3 RID-β	92.4	100.0
E3 14.7 kDa protein	100.0	99.3
U protein	100.0	98.1
fiber	99.7	98.8
E4 ORF 6/7 protein	100.0	100.0
E4 32 kDa protein	99.3	98.7
E4 ORF 4 protein	98.4	95.9
agnoprotein	99.4	97.0
E4 ORF 3 protein	99.1	99.1
E4 ORF 2 protein	96.9	98.4
E4 ORF 1 protein	98.4	96.0

**Table 3.** Protein percent identities of species F and G, relative to HAdV-G52. To assess the relationships between proteomes of species G and F, protein percent identities were calculated. One, denoted as “*” was not found in HAdV-G52.

Adenovirus	Percent Identity, relative to HAdV-G52														
	E1A	E1B 55 kDa protein	IVa2	DNA Pol	pTP	L1 55 kDa protein	L2 penton	L3 hexon	E2A DBP	CR1-alpha1 (RL1)	CR1-beta1 (RL2)	RL3*	L5 fiber1	L5 fiber2	E4 34 kDa

SAdV-G1	92.3	99.2	99.1	98.6	99.5	100	99.2	92.3	94.6	97.7	97.0	–	82.6	98.6	100
SAdV-G7	38.6	90.3	98.9	97.6	98.8	99.7	93.3	90.0	94.8	–	–	–	59.3	72.1	98.3
HAdV-F40	47.1	68.2	87.7	78.5	82.4	82.6	86.1	84.7	62.3	44.3	35.7	+	37.8	52.7	62.3
HAdV-F41	42.8	68.5	89.5	79.9	84.4	83.8	87.1	87.8	66.1	44.3	34.8	+	38.5	53.4	61.2
